# Supplementary material for: Evolving Notch polyQ tracts reveal possible solenoid interference elements
Source: PLoS One. 2017 Mar 20;12(3):e0174253. doi: 10.1371/journal.pone.0174253 (PMC5358852; doi:10.1371/journal.pone.0174253)
Supplement: S1 File — (DOCX) [file pone.0174253.s001.docx]

**File S1**

**Sequences used in alignment of polyQ tracts in fly NICD proteins**

**­­­ _┌———————— *Drosophila melanogaster*** vinegar fly Drosophilidae

**│ │_┌—————— *Glossina morsitans*** Tsetse fly Glossinidae Conceptual translation of CCAG010014983

**│ │_┌———— *Lucilia cuprina*** blow fly Calliphoridae AAC36153.1, AAC36151.1

**_│ |_┌—— *Musca domestica*** house fly Muscidae XP_011292982.1

**│ │ └—— *Stomoxys calcitrans*** stable fly Muscidae XP_013109509.1

**│ │_______┌—— *Ceratitis capitata*** fruit fly Tephritidae XP_004535280.1

**│ └—— *Bactrocera oleae*** fruit fly Tephritidae XP_014087559.1

**└———————————— *Anopheles darlingii*** mosquito Culicidae ETN64594, splice corrected from ADMH02000947

***D. mel.* KN**a**QsMQsL**---**Q**---------**gNg**L**D**MIKLD**NY**a**Ys**M**gspFQQ**E**LL**-**NgQg**L**g**M**NgNgQ**R**Ng**V**gpg**VL**pgg**Lc**g**M**gg**L**sg**a**gNgN**

*G. mor.* **KN**t**QsLQsLVp**-**Q**----------**ps**H**D**IIKLE**NYgYs**M**gspF**H**Q**E**LMNNgQsgNNs**a**pNsgN**VI**g**---------------------

*L. cup.* **KN**a**QsMQsLVp**-**QQ**----------**s**H**D**MIKME**NYgYs**M**gspF**H**Q**E**LMNgNQNgNNg**L**NgNN**MM**g**----------------------

*M. dom.* **KN**a**QsMQsLIp**-**QQ**----------**s**H**D**MIKME**NYgYs**M**gspF**H**Q**D**LMNgNpNNNsg**L**NgNN**VM**g**----------------------

*S. cal.* **KN**a**QsMQsLIp**-**QQ**----------**s**H**D**MIKME**NYgYs**M**gspF**H**Q**E**LMNsNQNgNNg**L**NgN**-L**g**-----------------------

*C. cap.* **K**tM**QsL**---**V**--**QQQQQQQQQQQNN**M**D**LIKME**NY**V**Ys**R**gspF**H**Q**E**ML**--**NQ**K**gNNQQs**M**N**tLc**ggggg**V**ggggpgN**L**g**H**sgpgN**M**g**

*B. ole.* **KN**a**Q**t**MQsLV**--**QQQQQQQQQQQNN**M**D**LIKME**NY**V**Ys**R**gspF**H**Q**E**MI**--**NQ**K**gNNQQs**M**Ns**Lc**ggggg**---**ggpgN**L**g**H**NgpgN**M**g**

*A. dar.* **Ks**aH**sIQsL**H**N**M**Q**------------H**D**-----**gYgYs**L**gNQF**aDL**LM**--**sQQQQQ**-----R**gg**V**N**a**pN**V**N**atatI**gsN**LVHtVMHa

***D. mel.***  **s**H**EQgLsppYsNQsppHsVQss**L**aLsp**Ha**Y**L**gspsp**a**Ks**R**psLptsptHIQaMRHa**t**QQKQ**

*G. mor.* **g**H**EQgLsppYsNQsp**-**HsVQsN**L**aLsp**Ha**Y**L**gspsp**a**Ks**R**psLptsptHMQaMRHa**t**QQKQ**

*L. cup.* **g**H**EQgLsppYsNQsppHsVQsN**M**aLsp**Ha**Y**L**gspsp**a**Ks**R**psLptsptHMQaMRHa**tH**QKQ**

*M. dom.* **g**H**EQgLsppYsNQsppHsVQsN**M**aLsp**Ha**Y**L**gspsp**a**Ks**R**psLptsptHMQaMRHa**t**QQKQ**

*S. cal.* **g**H**EQgLsppYsNQsppHsVQsN**M**aLsp**Ha**Y**L**gspsp**a**Ks**R**psLptsptHMQaMRHa**t**QQKQ**

*C. cap.* **Q**H**EQgLsppYsNQsppHsVQss**M**aLsp**Ha**Y**L**gspsp**V**K**tH**psLptsptHMQaMRHa**tH**QKQ**

*B. ole.* **N**H**EQgLsppYsNQsppHsVQss**M**aLsp**Ha**Y**L**gspsp**V**Ks**H**psLptsptHMQaMRHa**tH**QKQ**

*A. dar.* M**gEsgLsppYsNQsppHsVQs**tM**aLspQgY**I**gspsp**a**K**tR**psLptsptHIQaMRHaQ**H**QK**H

***D. mel.***  F**ggsNLNsLLgg**a**Nggg**VV**ggggggg**----**gg**V**gQ**-**gp**---------------**QNsp**V**s**L**g**II**s**

*G. mor.* Fc**gsNLN**t**LLgg**----------**ss**----a**sNg**V**NQNspNs**M**s**F**Q**t**sss**-----**Qssp**V**N**L**g**II**s**

*L. cup.* Fc**gNNLN**t**LLgg**----------**sg**----a**s**aL**QsQNspNs**M**N**F**Q**t**pss**-----**QNsp**V**s**L**g**II**s**

*M. dom.* Fc**gsNLN**t**LLggggN**t**NgNg**Va**sg**----a**s**tM**Q**t**QNspNs**M**N**F**Q**t**pss**-----**QNsp**V**s**L**g**II**s**

*S. cal.* Fc**gNNLN**t**LLgg**------**sg**V**gsg**----a**s**tL**QsQNspNs**M**s**F**Q**t**pss**-----**QNsp**V**s**L**g**II**s**

*C. cap.* F-**gNNVNsLLgg**aa**NN**t**NsNs**t**NgpQsN**a**s**----M**NspQs**M**s**F**QspssQs**L**sQQNsp**V**s**V**g**IV**s**

*B. ole.* F-**gNNVNsLLgg**aV**NN**a**NsNs**t**NgpQsN**a**s**----M**NspQs**M**s**F**QspssQs**L**sQQNsp**V**s**V**g**IV**s**

*A. dar.* **NN**KMI**sNgNL**--**QQQQQQQQ**La**s**t**s**LL**gssggNNg**a--**N**LML**Ngss**ML**gg**L**Q**I**QN**F--------

***D. mel.***  **ptgs**D**Mg**I**ML**a**ppQ**----------------------------**ss**--------

*G. mor.* **ptgs**D**Mg**I**ML**tt**QQQ**I**QQQQQQQQQQQQQ**L**QQQQQQQ**H**QQ**----**NNN**-----

*L. cup.* **ptgs**D**Mg**I**MM**aa**NQQQQQQ**L**QQQQQQQQQQQQ**-------------**N**------

*M. dom.* **ptgs**D**Mg**I**MM**a**sQQQQQQQQQ**--M**QQQQQQQQ**-------------**N**------

*S. cal.* **ptgs**D**Mg**I**MM**a**sQQQQQQQQQQQ**M**QQQQ**-----------------**N**------

*C. cap.* **ptgs**D-**g**M**MM**a**pQQ**t**QQQQQQQQQQQ**L**QQQQQQQQ**H**QQQQQQQQQNs**-----

*B. ole.* **ptgs**D-**g**M**MM**a**pQQ**t**QQQQQQQQ**L**QQQQQQ**H**QQ**H**QQQ**--------**Ns**-----

*A. dar.* V**tgss-g**LE**L**a**g**Fc**sp**t**gQgsQ**a**s**t**sQg**Va**gNNsg**aM-----**sssNspg**Mat

***D. mel.***  K**Ns**aI**MQ**t**IspQQQQQQQQQQQQQ**H**QQQQQQQQQQQQQQQQQ**-----------L**gg**---------LEF**gs**a**gL**DL**Ng**Fc**gsp**...

*G. mor.* K**ss**aI**LQ**t**M**--**QQQ**HHH**QQQQQQQQQQQQ**------------------------L**ggg**--**N**a**gsN**-IEF**ss**a**gL**DL**Ns**Fc**gsp**...

*L. cup.* K**Ns**aI**MQsM**--**QQQQQQQ**-----------------------------------M**gg**-**NNNggpg**-MDF**ss**a**gL**DL**Ns**Fc**gsp**...

*M. dom.* K**Ns**aI**LQsM**--**QQQQQQQQQQQQQ**-----------------------------M**g**--**NNNggggg**IDF**ss**a**gL**DL**Ns**Fc**gsp**...

*S. cal.* K**Ns**aI**LQsM**--**QQQQ**H**QQ**-----------------------------------M**ggN**t**NNggg**--MDF**ss**a**gL**DL**Ns**Fc**gsp**...

*C. cap.* K**ss**aI**MQ**t**M**--**QQ**H**QQQ**------------------------------------La**s**---------LDF**g**ta**gL**DL**Ng**Fc**gsp**...

*B. ole.* K**ss**aI**MQ**t**M**--**QQ**H**QQQ**------------------------------------L**gs**---------LDF**gs**a**gL**DL**Ng**Fc**gsp**...

*A. dar.* a**p**tHa**LspL**t-**QQQQQQ**VLVMVa**QQQ**a**Q**L**NNQ**H**QQQQ**L**QQQ**L**Q**H**Q**H**QQQQ**HH**Q**H**QQQQ**H**-Q33-**I**gQQpQQQLgQQQQQpQQ**...
