# Supplementary material for: Evolving Notch polyQ tracts reveal possible solenoid interference elements
Source: PLoS One. 2017 Mar 20;12(3):e0174253. doi: 10.1371/journal.pone.0174253 (PMC5358852; doi:10.1371/journal.pone.0174253)
Supplement: S2 File — (DOCX) [file pone.0174253.s002.docx]

**File S2**

**Full-length alignment of NICD for fly, bee, and human Notch proteins**

S3

**Fly Notch ^** MV**Ls**t**QR**K**R**a**Hg**Vt**WFpE
Bee Notch ^** VLVta**QR**K**R**aV**g**Vt**WFpE**

**Human Notch1 ^** VL**Ls**RK**R**R**RQHgQ**L**WFpE**

**gFR**a**p**a**a**VM**s**R**RR**R----d**p**H**gQ**EMR**NLN**K**Q**V-**a**M**QsQg**-V**gQpg**aH-**Ws**d**dEsd** **<** RAM
 **gFL**Rt**ssgsgQRR**R**s**RRR**gp**d**gQ**EMR**N**L**N**K**Qps**V**N**cMdLdV**gNg**Ra**QQ**W**s**ddE**s**d

**gFK**V**s**E**as**KKK**RR**-----E**p**L**g**Ed**s**V**gL**K**p**LK**Nas**d**g**aLMdd**NQN**E--**Wg**-**dE**-**d**

M**p**--**KRQRs**-d**pVsg**V**gLgNNgg**Ya**sd**H**t**MV**s**EYEEa **< NLS**
 **LppsKRMR**aIE**p**----------**g**Ya**sd**H**t**aItdYEEt

**L**E--**KKFR**FEE**pV**VL**p**d**L**-------d**dQt**--------

**dQR**V**WsQ**a**HLd**VV**d**V**R**a--I**M**--**tpp**-aH**Qdgg**KH**dV**da**Rgp**L**p** E**pR**M**W**t**QQHLdaa**EI**R**R**p**d**ag**VL**tpps**LEH---**gQdV**da**Rgp**--

**d**H**RQW**t**QQHLdaad**L**R**M-**saM**a**ptppQg**EV**d**adcM**dVN**V**Rgp**-t

c**g**L**tpLMIaa**VR**gggL**d**tg**EdI**ENNEds**t**aQVIsdLL**a**Qga**E**LN**atM**d**K **<** **aR-1**

c**g**M**tpLM**V**aa**VR**gggL**d**tg**EE-**E**d**Esdg**t**a**a**VI**a**dLV**a**Qga**d**LN**at**td**K

d**g**F**tpLMIas**c**sgggL**E**tgNs**-**E**E**EEd**a**pa**-**VIsdFI**Y**QgasL**H**NQtd**R

**tgEtsLHLaaR**F-----------**aR**----a**daaKRLLdagadaN**c**QdN < aR-2**

**sgEtsLHLaaRY**-----------**aR**----a**daaKRLLdagadaNsQdN**

**tgEt**a**LHLaaRY**-----------**sR**----**sdaaKRLL**E**asadaN**I**QdN**

t**gRtpLHaaV**a**a**-----------**da**----M**gVFQILLRNRatNLNaRMH < aR-3**

t**gRtpLHsaV**a**a**-----------**da**----M**gVFQILLRNRatNLNaRMH**

M**gRtpLHaaVsa**-----------**da**----**QgVFQILIRNRat**d**L**d**aRMH**

**dgttpLILaaRL**-----------**a**I----**EgMVEdLI**tad**ad**I**Na**a**dN < aR-4**

**dgttpLILaaRL**-----------**a**t----**EgMVEdLIN**ad**adINa**a**dN**

**dgttpLILaaRL**-----------**a**V----**EgM**L**EdLINs**H**ad**V**Na**V**d**d

**sgKtaLHWaaaV**-----------**NN**----tE**aVN**I**LL**MHH**aN**R**d**a**Qd**d **<** **aR-5**

**sgKtaLHWaaaV**-----------**NN**----**V**d**aVN**I**LL**VH**gaN**R**d**a**Qd**d

**LgKsaLHWaaaV**-----------**NN**----**Vda**aVV**LL**K**NgaN**K**d**M**QNN**

Kd**EtpLFLaaRE**-----------**gs**----**YEa**c**K**a**LLdNFaNR**E**ItdH < aR-6**

Kd**EtpLFLaaRE**-----------**gs**----F**Ea**c**K**a**LLd**t**FaNR**E**ItdH**

RE**EtpLFLaaRE**-----------**gs**----**YE**ta**K**V**LLd**H**FaNR**d**ItdH**

**MdRLpRd**V**asER**-----------L**H**----**HdIV**-**RLLdE**HV-**pRspQ**M **<** **aR-7**

**MdRLpRd**V**asER**-----------L**H**----**HdIV**-**RLLdE**HV-**pRspQ**M

**MdRLpRd**I**aQER**-----------M**H**----**HdIV**-**RLLdE**Y**N**LV**RspQ**L

L**s**Mt**pQ**-aMI**g**----------**sppp**----**gQQQpQLI**t**Qp**tVI**s**a**gNg** **<** **aR-8?**

V**N**VI**pNgp**LM**g**----------**spN**--------H**pQLI**tH**p**tVI**gs**a**p**K

H**g**-a**pLgg**t**p**tL---------**spp**-----Lc**spNgYL**--**gs**LK**pg**V**Qg**

**gNNgNgN**a**sg**K**QsNQ**taK**Q**Kaa**KKaKL**IE **<** NLS2

------------------**Q**aK**sKKRpK**a**g**

----------------------**KKVRKps**

-----a**gsp**d**Ng**-**L**d**a**t**gs**L**RRK**a**ss**K-**K**t**s**aa**s**KKaa**NLNgLN**-**pgQL**t**gg**V**sg**V**pg**V**pp**t**Ns**aa**Q**

**s**t**gN**-**pNspE**-**s**E-**gg**VVVV**RRKps**VKK-----------**pp**aKR**g**a**Q**------------**ppNQ**EI**pQ**

**s**K**g**Lac**gs**K**E**aKd**L**K**a**----**RRK**K**sQ**d**gKg**--------c**L**--**L**d**ssg**M**L**------------------

aaaaaaaVaaM**s**HELE**gspVg**V**g**M**ggN**L**psp**Ydt**ss**MY**sN**aMaa**p**La**NgNpN**t**g**a**KQppsY**Edc**I** **< Nedd.**

-------------------**g**aE**g**aE**gN**L**psp**Yd**s**a**s**LY**sN**aI--**p**LV-**g**Htat-a**KQpppY**Edc**I**

-----------------**spV**--------------------------------d**s**LE**sp**H**gY**---**L**

**KN**a**QsMQsLQgNgLd**MIKLd**NY**aY**s <** **pQ slot A**

**KgQ**-**sMQgLQQ**L**gLd**tFt-t**NY**---

**s**dVa**spp**-**L**----------------

M**gspFQQ**E**LL**-**NgQgL**-**gMNgNgQRNgVgpg**-**V**L**pgg**-**L**c**gMgg**L**sgagNgN <** **pQ slot B**

**g**L**pNF**Hd**QLL**a**s**H----------**QRQ**------------------------a**Q**

L**pspFQQsps**V**p**L**N**H**LpgMp**-dtHL-**gIg**HL**NV**aaK**p**E**M**aa**Lgg**-**gg**RLaFE

**s**HE**QgLsppYsNQsppHsVQss**--**LaLsp**H**a**-**YLgspspaKsRpsLp <** **pQ slot C**

**g**MV**N**t**LsppYsNQsppHsVQsN**Mt**L**--**spQasYMgspspaKsRpsLp**

t**gpp**R**Ls**H**Lp**Va**sg**t**s**tV**LgsssggaLN**F--t**Vggs**-----------

**tsptHIQaMR**H**atQQKQ <** **pQ slot D**

**tsptHI**a**aMRQat**H**QK**H

**ts**L**NgQ**cE**WLs**RL**Qsg**-

F**ggsNLNsLLgg**-**aNggg**VV**ggggggggg**V**g**-**QgpQNsp**V**s**L**g**II**s <** **pQ slot E**

**gg**M**p**-----------------------------------------
MV**pNQYNpL**R**gs**V**apgp**------------L**s**t**Q**a**p**----------**s**

**p**t**gs**d**Mg**I**M**---**LappQss**---------------- **<** **pQ slot F**

**p**V**g**Fd**F**E**NL**---**p**Y**sssQQQQQQQQQQQQQQQQQQ**
L**Q**H**g**M**VgpL**H**ssLa**a--**s**aL---------------

K**Ns**aI**MQtIsp**-----**QQQQQQQQQQQQQ**H-**QQQQQQQQQQQQQQQQQLgg**-------- **<** **pQ slot G**

**MQQ**t**QQQ**I**QQQQQQQQQQQQQQQQ**

**sQ**MM**sYQgLps**tRLat**Qp**HLV**Q**t**QQ**V**QpQN**L**Q**M**QQQN**L**Qp**a**N**I**QQQQsLQppppppQp**H

**L**E**Fgsa**---**gL**dL**NgF**c**gsp**d**s**FH**sgQMNppsIQs**--**sMsgssps**t**N**M-**LspssQ**H**NQQa**FY

-----------------------------------------**s**t**QQQQQ**a**QQQQ**a**QQQ**t**QQ**YY

**LgVssaasg**H**Lg**R**s**F**Lsg**E**psQ**adV**QpLgpssL**aVHtI**LpQ**E**sp**aL**p**t**sLpss**LV**pp**Vt**a**-a

**Q**Y**LtpssQHsgg**----H**tpQH**-**L**V**Q**tLd-**sYptpspEspg**H**Wssssp**R**sN**-**sdWsEgVQsp <** DUF3454

**Q**Y**Ltpps**H**Hsgp**d---V**tpQH**-**L**M**Q**a**pE**-**sFptpsp**d**spg**H**WsssspHsN**-**sdWsE**cMa**sp**
 **Q**F**LtppsQHs**Y**ssp**Vd**NtpsHQL**-**Q**V**pE**H**pF**L**tpspEspdQWsssspHsN**V**sdWsEgVssp**

aa**NN**LYI**sgg**H**Q**a**N**K**gsEa**I**Y**I*****

--**N**aY**g**a**g**aaH**QsN**K**gsEa**I**Y**I*****

-----**p**t**s**M**QsQ**IaRI**pEa**-**F**K*****
